# Clinical features of patients with carcinoma soft tissue metastases as surgical indications: a retrospective cohort study

**DOI:** 10.1186/s12885-024-12350-2

**Published:** 2024-05-10

**Authors:** Sei Morinaga, Norio Yamamoto, Katsuhiro Hayashi, Akihiko Takeuchi, Shinji Miwa, Kentaro Igarashi, Yuta Taniguchi, Yohei Asano, Takayuki Nojima, Hiroyuki Tsuchiya

**Affiliations:** 1 Department of Orthopedic Surgery, Graduate School of Medical Sciences, Kanazawa University, 13-1 Takara-machi, Kanazawa, Ishikawa 920-8640 Japan; 2 Department of Diagnostic Pathology, Kanazawa University Hospital, 13-1 Takara-machi, Kanazawa, Ishikawa 920-8640 Japan

**Keywords:** Soft tissue metastasis, Carcinoma, Diagnosis, Surgical indication

## Abstract

**Background:**

Soft-tissue metastasis of carcinoma is rare. In the present study, we investigated the surgical indications and clinical features of patients with soft tissue metastases of carcinoma.

**Methods:**

In this retrospective cohort study, we enrolled 26 patients with soft tissue carcinoma metastasis referred to our department for treatment. Sex, age, location, size, depth, pain due to the tumor, primary origin, serum C-reactive protein (CRP) level, MRI examinations, diagnosis by a previous physician, carcinoma markers from blood, history of carcinoma, other metastases, performance status (PS), and surgical procedures were documented. Associations between variables and surgery were statistically analyzed.

**Results:**

The primary cancer origin was found to be the lung (*n* = 10), kidney (*n* = 7), esophagus (*n* = 2), stomach (*n* = 1), breast (*n* = 1), liver (*n* = 1), ureter (*n* = 1), anus (*n* = 1), and unknown (*n* = 2). The mean CRP level of all patients was 2.3 mg/dL. Seven tumors (26.9%) were originally suspected to be soft tissue metastases of carcinoma, while 19 tumors (73.1%) were considered soft tissue sarcomas or inflammatory lesions by the previous treating physician. Twenty patients (76.9%) had other metastases. The PS of the 12 patients (46.2%) was zero. Eleven patients (42.3%) underwent surgery for soft tissue metastases. Diagnosis of soft tissue metastasis by a previous physician and good PS (*p* < 0.05) were significantly associated with surgery.

**Conclusion:**

Overall, the present results show that surgical indications for soft tissue metastasis of carcinoma include diagnosis by the referring physician or good PS of the patients.

## Background

Soft-tissue metastasis of carcinoma is rare; a previous study described the prevalence of soft tissue metastasis in patients with solid malignancy to be only 0.2–1.2% [1, 2]; however, a separate autopsy series study identified a 16–17.5% prevalence of soft tissue metastasis [3, 4]. In 2022, 1,918,030 new cancer cases were projected to be diagnosed in the United States [5]. As of January 1, 2022, more than 18 million Americans were living with a history of cancer [6]. As advances in medicine continue to progress, the number of cancer survivors is expected to increase; hence, the prevalence of soft tissue metastasis of carcinoma is expected to increase accordingly.

Patients with soft tissue metastasis have a poor prognosis, with a one-year survival of 25–55%, and a two-year survival of only 0–33% [7, 8]. Additionally, reports have described that some patients with soft tissue metastasis are commonly initially misdiagnosed as having soft tissue sarcoma or an inflammatory lesion [7–9]. Hence, accurate diagnosis and proper treatment are of utmost importance in patients with soft tissue metastases. Once the diagnosis of soft tissue metastasis of carcinoma is made, a treatment plan should be promptly designed in cooperation with the department in charge of the primary organ.

Currently, there are limited studies investigating the surgical indications for soft tissue metastases of carcinoma. Therefore, in the present study, we investigated the surgical indications and clinical features of patients with soft tissue metastasis of carcinomas.

## Materials and methods

This non-controlled, retrospective analysis was conducted using the data obtained from the medical records of a single institution. This study was approved by the Ethics Committee of our hospital, and informed consent was obtained from all patients. Patients referred to our department and diagnosed with soft tissue carcinoma metastasis between January 2007 and December 2021 were identified and enrolled. Patients with lymph node metastasis or hematologic cancer, such as malignant lymphoma, were excluded. After application of these criteria, 26 patients were included in the study.

We collected the demographic and clinical features of all participants, including sex, age, location, size, depth, pain due to the tumor, primary origin, serum C-reactive protein (CRP) level (Normal range: 0-0.3 mg/dL), magnetic resonance imaging (MRI) examinations (T1-weighted sequence, T2-weighted sequence, and contrast-enhanced MRI), diagnosis of the previous physician, carcinoma markers from blood, history of carcinoma, other metastases, performance status (PS), and surgery for soft tissue metastasis. Tumor depth was defined as superficial or deep in relation to the investing muscular fascia.

All statistical analyses were performed using EZR (Saitama Medical Center, Jichi Medical University, Saitama, Japan). Associations between the variables and surgery for soft tissue metastasis were statistically analyzed using Fisher’s exact test. Statistical significance was set at *p*-values < 0.05 for all other analyses.

## Results

Twenty-six patients, including 22 men and four women, were enrolled in the present study. The mean patient age was 68.6 years (range: 46–93 years). Four metastases were located in the upper extremities, nine in the lower extremities, and 13 in the trunk. The mean metastasis size was 5.2 cm (2.1–13.9 cm). Twenty-four metastases (92.3%) were located beneath the muscle fascia. Thirteen patients (50%) experienced pain due to the metastasis. The primary origin of cancer was the lung in 10 patients, kidney in seven, esophagus in two, stomach in one, breast in one, liver in one, ureter in one, anus in one, and unknown in two. The mean CRP level was 2.3 (0.1–13.6) mg/dL. Seven metastases (26.9%) were suspected as soft tissue metastases of carcinoma, and the remaining 19 (73.1%) were believed to be soft tissue sarcoma or inflammatory lesions by the previous physician. Carcinoma markers from blood were positive in 13 patients (CYFRA in four, CEA in three, SCC in three, PSA in three, CA15-3 in two, ProGRP in one, CA19-9 in one, CA125 in one), negative in six, and unknown in seven. Sixteen patients (61.5%) had a history of carcinoma, 20 patients (76.9%) had other metastases, and the PS of the 12 patients (46.2%) was zero. Overall, 11 patients (42.3%) underwent surgery for soft tissue metastases (Table 1).

**Table 1:** Characteristics of the study participants

Characteristic		Number
Sex	Male	22
	Female	4
Mean age		68.6 (46–93)
Location	Upper extremity	4
	Lower extremity	9
	Trunk	13
Mean tumor size (cm)		5.2 (2.1–13.9)
Depth	Superficial	2
	Deep	24
Pain	+	13
	-	13
Primary origin	Lung	10
	Kidney	7
	Esophagus	2
	Stomach	1
	Breast	1
	Liver	1
	Ureter	1
	Anus	1
	Unknown	2
Mean CRP		2.3 (0.1–13.6)
Diagnosis at previous doctor	Soft tissue metastasis	7
	Others	19
Carcinoma marker	+	13
	-	6
	Unknown	7
History of carcinoma	+	16
	-	10
Other metastasis	+	20
	-	6
PS	0	12
	≧ 1	14
Surgery for soft tissue metastasis	+	11
	-	15

CRP; C-reactive protein

**Fig. 1:**
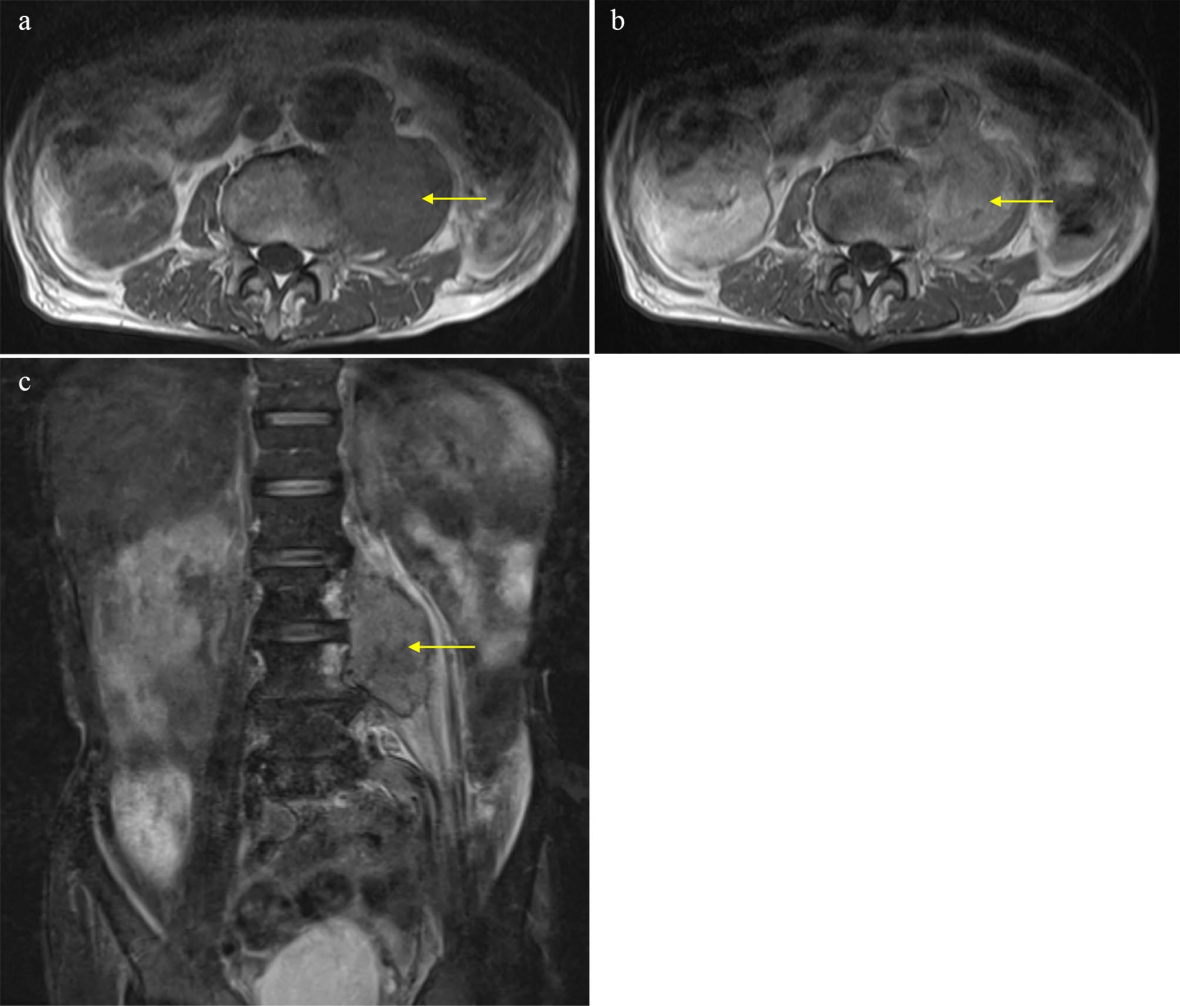
MRI results. (**a**) axial T1-weighted, homogeneous iso-intensities; (**b**) T2-fat suppression, heterogeneous high-intensities; (**c**) axial enhanced, heterogeneous-enhanced lesion

Of the 26 patients enrolled, 22 underwent MRI, of whom 11 underwent contrast-enhanced MRI. The T1 signals of the 21 metastases (95.4%) presented as homogeneous iso-intensities, while on T2 imaging, 17 metastases (77.3%) were heterogeneous high-intensities. Ring-enhanced lesions were confirmed in five patients (Fig. 1), heterogeneous enhanced lesions in five, and homogeneous enhanced lesions in one (Table 2).

**Table 2:** MRI characteristics of the study participants

Characteristic	Intensity finding	Number
MRI (T1)	Homogeneous iso	21
	Heterogeneous low and high	1
	Unknown	4
MRI (T2)	Heterogeneous high	17
	Heterogeneous low and high	3
	Homogeneous	2
	Unknown	4
contrast-enhanced MRI	Ring-enhanced	5
	Heterogeneous enhanced	5
	Homogeneous enhanced	1
	Unknown	15

MRI; Magnetic resonance imaging

Fisher’s exact test confirmed that a previous diagnosis of soft tissue metastasis and good PS (*p* < 0.05) was significantly associated with surgery for soft tissue metastasis (Table 3).

**Table 3:** Correlation between surgery for soft tissue metastasis and patient variables

Variables			Surgery for soft tissue metastasis		Fisher’s exact test
		n	+	-	*P* value
Sex	Male	22	8	14	0.28
	Female	4	3	1	
Age	<65	9	4	5	1
	≧ 65	17	7	10	
Location	Extremity	13	5	8	1
	Trunk	13	6	7	
Size (cm)	< 5	15	8	7	0.25
	≧ 5	11	3	8	
Depth	Superficial	2	2	0	0.17
	Deep	24	9	15	
Pain	+	13	5	8	1
	-	13	6	7	
CRP	<0.4	10	5	5	0.7
	≧ 0.4	15	6	9	
Diagnosis at previous doctor	Soft tissue metastasis	7	6	1	< 0.05
	Others	19	5	14	
Carcinoma marker from blood	Positive	13	4	9	0.62
	Negative	6	3	3	
	Unknown	7			
History of carcinoma	+	16	8	8	0.43
	-	10	3	7	
Other metastasis	+	20	6	14	0.05
	-	6	5	1	
PS	0	12	8	4	< 0.05
	≧ 1	14	3	11	

In an example case, an 81-year-old man presented with a 3-month history of lower back pain but no swelling documented at a previous hospital. The patient had a medical history of abdominal aortic aneurysm and surgery for ureter cancer five years prior. Magnetic resonance imaging (MRI) revealed a mass in the left iliopsoas muscle (Fig. 1).

**Fig. 2:**
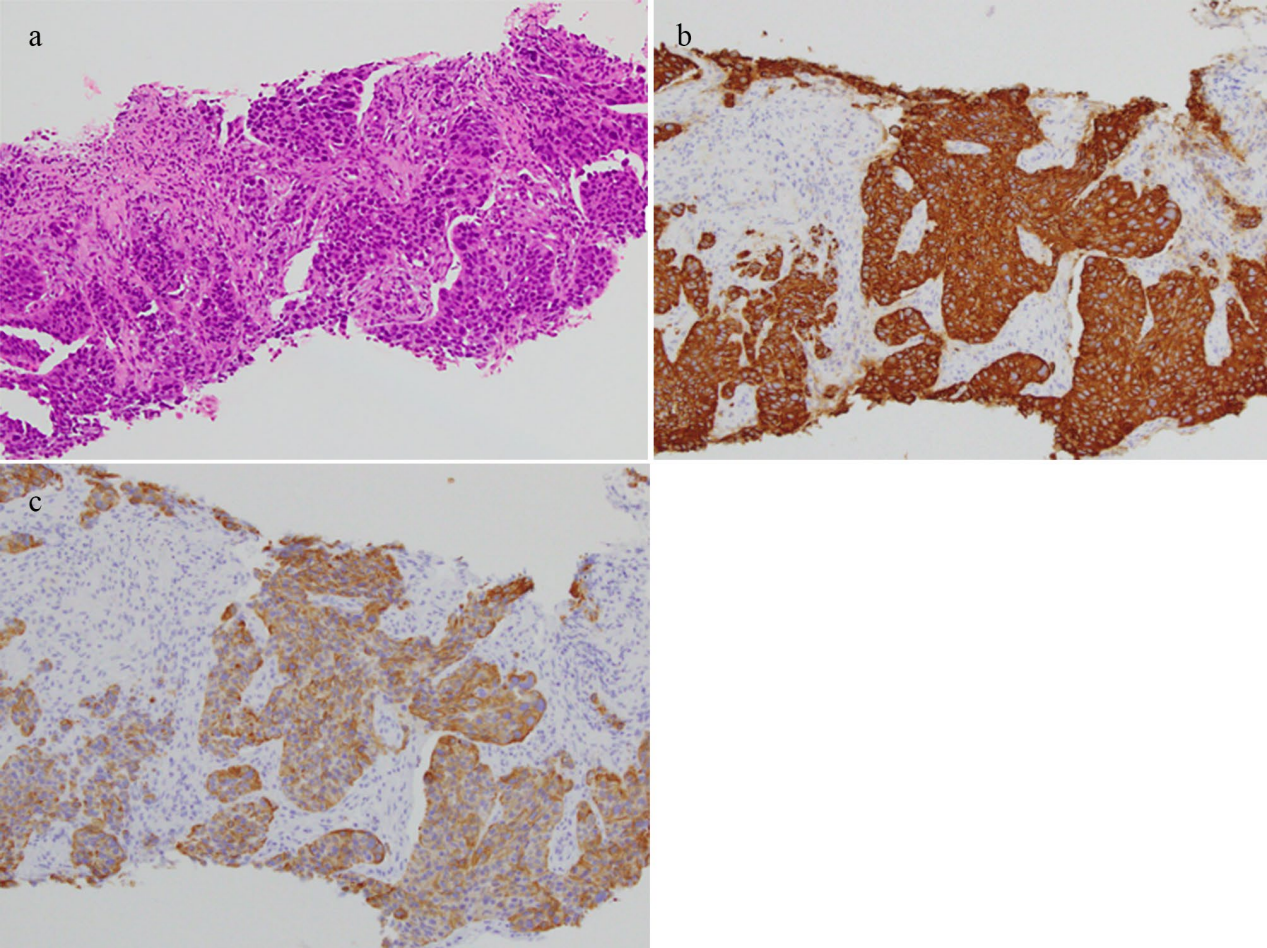
Histological results of the needle biopsy indicated metastasis of urothelial carcinoma (**a**) Hematoxylin eosin staining. The biopsy sample shows positive staining with (**b**) cytokeratin (CK) 7; and (**c**) CK 20

The T1 signal of the lesion was homogenous iso-intensity, and T2-fat suppression was heterogeneous high-intensity. Contrast-enhanced MRI revealed that the lesion was heterogeneously enhanced. The patient was diagnosed with a left iliopsoas abscess by a previous doctor, and was treated with intravenous antibiotics. However, the patient showed no notable improvement and was subsequently referred to our department. The histological results of the CT-guided biopsy revealed soft tissue metastasis from the ureteral cancer (Fig. 2). Surgery for soft tissue metastases was not performed.

## Discussion

Soft tissue metastases of carcinomas are rare. Prior studies have reported that blood flow is unstable in the skeletal muscles, and tumor cells are destroyed by physical muscle stimulation as lactic acid invades tumor cells [10, 11]. Hence, tumor growth rarely occurs in such environments. Unfortunately, there have been relatively few studies with a large number of patients investigating this topic [1, 2, 8, 10, 12–14], and as such, there are no guidelines for surgical indications. Therefore, in the present study, we investigated the surgical indications and clinical features of patients with soft tissue metastasis of carcinomas.

Soft tissue metastases from carcinoma were frequently observed in the trunk area in previous large case series [2, 13], while the existing literature describes the mean tumor size as 5–8.5 cm [1, 9, 14, 15]. Moreover, lung cancer is the most common primary carcinoma, followed by kidney and colon cancers [8, 13]. In the present study, soft tissue metastases were most common in the trunk, the mean tumor size was 5.2 cm, and the most frequent primary origin was the lungs. Previous reports have described pain as a frequent symptom [1, 7, 10, 14, 16, 17], and research has further suggested that soft tissue metastasis of carcinoma may induce pain owing to inflammation [14].

The CRP level reflects the inflammatory cytokines produced by cancer cells and the host immune response to cancer cells [18]. Thirteen patients (50%) experienced pain due to the tumor, and the mean CRP level was 2.3 mg/dL in this analysis. In addition, Ishibashi et al. [15] reported that an elevated pretreatment CRP value increased to 0.4 mg/dL or above was an independent predictor of poor prognosis in patients with soft tissue metastasis. Therefore, CRP estimation can help surgeons decide whether to pursue radical surgery. In the present analysis, the number of patients with CRP levels > 0.4 mg/dL was 15, and surgical excisions were performed for six of these patients.

MRI for soft tissue metastasis of carcinoma predominantly reveals lesions with poorly defined margins, low T1-signal intensity, high T2-signal intensity, and enhancement with gadolinium [19, 20]. Tuoheti et al. [1] reported that the infiltrative borders of the tumor should be excised to the greatest extent possible after the excision area has been predetermined according to the peritumoral area on the MR image. In our series, wide excisions were performed in all surgical cases. In addition, strong enhancement around the tumor with gadolinium was considered to indicate central necrosis, which has been shown to be a characteristic feature of soft tissue metastasis of carcinoma [1, 12]. Moreover, MRI with gadolinium for soft tissue abscesses showed ring-enhanced lesions [21] and heterogeneous enhancement on MRI with gadolinium has been linked to soft tissue sarcoma [22]. In the present analysis, 73.1% of the patients with soft metastasis of carcinoma were suspected of having a soft tissue sarcoma or an inflammatory lesion by a previous doctor. Soft tissue metastasis of carcinoma can be mistaken for soft tissue abscess and sarcoma, similar to the case described in this study. Accurate diagnosis of soft tissue metastasis of carcinoma is paramount, as its treatment and prognosis significantly differ from those of other diseases. Determining the organ of origin of metastatic carcinoma should be promptly performed through assessment of clinical information, such as the past history of carcinoma, status of the past carcinoma, radiological findings, and histological results.

An increase in the number of cancer survivors due to advances in medical care has contributed to an increasing number of soft tissue metastases from carcinoma. Tuoheti et al. [1] previously described the cases of four patients with soft tissue metastasis during a seven-year period (1994–2001), whereas eight patients with soft tissue metastasis were found during the next two years (2001–2003). This sudden increase in patients with soft tissue metastasis within two years may be related to the increased incidence of carcinomas due to aging and other factors. In our analysis, seven patients with soft tissue metastasis of carcinoma were detected during a seven-year period (2007–2013), and 19 patients were detected during an eight-year period (2014–2021).

Although guidelines for bone metastasis of carcinoma exist, there are no standardized treatment guidelines for soft tissue metastasis of carcinoma owing to its rarity. Most soft tissue metastases of carcinoma are detected incidentally during the staging examination [2]. After detection, treatment of soft tissue metastases depends on the clinical setting in relation to the primary disease (concurrent lesions or recurrence after treatment of primary disease), the symptoms and general condition of the patient, and the histological type and extent of the metastasis (local or disseminated disease) [23]. In the present analysis, surgeries for soft tissue metastases of carcinoma were performed significantly more often for tumors diagnosed as soft tissue metastasis by the previous physician and patients who had good PS. Studies on cancer have indicated that poor preoperative PS was associated with postoperative complications and morbidities [24–27], which implies that patients with poor PS have worse outcomes when undergoing surgery. The results of our study indicate that surgery is more commonly indicated if there is a request for resection from the attending physician of the primary cancer department or if patients have good PS.

Our study has several limitations. First, this was a retrospective study, which would have introduced inevitable bias. Second, this study had a small sample size and multivariate analysis was not applied. Lastly, an analysis of the prognosis of soft tissue metastasis of carcinoma was not conducted. However, the life expectancy of patients with carcinoma depends on the type, and bias was considered.

In summary, this study analyzed the clinical features of soft tissue metastases from carcinoma. Surgical indications include a request for resection from the attending physician of the primary cancer department or good PS of the patients.

## Data Availability

The datasets supporting the conclusion of this article are included within the article. The underlying datasets are available from the corresponding author on reasonable request.
